# Antimicrobial consumption in an acute NHS Trust during the COVID-19 pandemic: intervention time series analysis

**DOI:** 10.1093/jacamr/dlae013

**Published:** 2024-02-07

**Authors:** Sidra Khan, Stuart E Bond, Jade Lee-Milner, Barbara R Conway, William J Lattyak, Mamoon A Aldeyab

**Affiliations:** Department of Pharmacy, School of Applied Sciences, University of Huddersfield, Huddersfield, HD1 3DH, UK; Department of Pharmacy, School of Applied Sciences, University of Huddersfield, Huddersfield, HD1 3DH, UK; Pharmacy Department, Mid Yorkshire Teaching NHS Trust, Wakefield, WF1 4DG, UK; Pharmacy Department, Mid Yorkshire Teaching NHS Trust, Wakefield, WF1 4DG, UK; Department of Pharmacy, School of Applied Sciences, University of Huddersfield, Huddersfield, HD1 3DH, UK; Institute of Skin Integrity and Infection Prevention, University of Huddersfield, Huddersfield, HD1 3DH, UK; Statistical Consulting Department, Scientific Computing Associates Corp., River Forest, IL 60305, USA; Department of Pharmacy, School of Applied Sciences, University of Huddersfield, Huddersfield, HD1 3DH, UK

## Abstract

**Objective:**

To determine the impact of the COVID-19 pandemic on antimicrobial consumption and trends of therapeutic drugs for COVID-19 treatments, including corticosteroids, remdesivir and monoclonal antibodies (tocilizumab) from April 2017 to September 2022 in a secondary care NHS Trust in England.

**Methods:**

A retrospective intervention time series analysis was conducted for April 2017 to September 2022 at the Mid Yorkshire Teaching NHS Trust. Data were retrieved from the pharmacy dispensing system as defined daily doses (DDDs) monthly and reported per 1000 occupied bed days (OBDs). Antimicrobial consumption and COVID-19 treatment options were measured. DDDs were calculated according to the classification of antimicrobials for systemic use (J01) and for other drugs classification. Trends for antimicrobial consumption and other therapeutic drugs for treating COVID-19 were also determined in each wave in England.

**Results:**

During the pandemic: total antibiotic consumption decreased from 826.4 to 728.2 DDDs per 1000 OBDs (*P* = 0.0067); piperacillin/tazobactam use increased (*P* < 0.0001) and ciprofloxacin use decreased (*P* < 0.0001); there were no changes in Access, Watch, Reserve antibiotic use, and the proportion of antifungal consumption was consistent throughout the study. The use of total antibiotics (*P* = 0.024), levofloxacin (*P* = 0.0007), piperacillin/tazobactam (*P* = 0.0015) and co-amoxiclav (*P* = 0.0198) increased during wave one. Consumption of COVID-19 treatment drugs was highest during wave two, with 624.3 DDDs per 1000 OBDs for dexamethasone (*P* = 0.4441), 6.8 DDDs per 1000 OBDs for remdesivir (*P* < 0.0001) and 35.01 DDDs per 1000 OBDs for tocilizumab (*P* = 0.2544).

**Discussion:**

This study determined the consumption of antimicrobials trends before and during the pandemic. The individual wave antimicrobial consumption indicates maximum consumption in the first wave, advocating for antimicrobial stewardship and preparedness for future pandemics.

## Introduction

Since the beginning of the COVID-19 pandemic, increased antibiotic consumption has been a subject of attention and consequence in an upsurge of antimicrobial resistance (AMR).^1^ Published studies have shown only 4%–15% confirmed secondary bacterial infections in hospitalized patients with COVID-19 and are substantially associated with greater mortality.^2–4^ An additional study from the USA and India reported that a considerable increase in antibiotic consumption was observed in the first two waves of the pandemic; a drop in antibiotic consumption was noted later.^5,6^ However, a multicentre study by the International Severe Acute Respiratory and Emerging Infections Consortium (ISARIC), World Health Organization (WHO) Clinical Characterization Protocol UK (CCP-UK), revealed that 85% of inpatients with COVID-19 received at least one antibiotic agent during the first wave. In the UK-based study, the prescribing trend increased from 2017 to September 2020, including the pandemic period from February to September 2020. This study also found that 11% of suspected or confirmed patients with COVID-19 received antibiotics.^7^ Due to the absence of clear national guidelines and irrespective of disease severity, empirical antibiotic therapy was widespread during the initial phase of the pandemic.^8–12^ Moreover, a meta-analysis of data from high-income countries (HICs) and low-middle-income countries (LMICs) showed antibiotics were used in 68% of patients with COVID-19. Sub-group analysis found that 58% of patients were prescribed antibiotics in HICs and 89% in LMICs.^13^ This raises concerns about the impact of COVID-19 on empirical antibiotic therapy, withdrawal of antimicrobial stewardship (AMS) activities and enhanced risk of AMR.^14–18^ Irrational use of antimicrobials also resulted in adverse drug events, and affected patient safety and quality of care resulting in increased healthcare costs.^1^ Several studies from secondary healthcare settings in the UK reported increased antibiotic consumption with no evidence of bacterial infection.^19–22^ A recent study found mixed views regarding AMS activities carried out during the pandemic in the UK reporting that AMS was not a priority during the height of the pandemic, which could affect overall antibiotic use during this time.^23^

The trends of antimicrobial consumption were analysed by using the intervention time series analysis (ITSA) method. A time series is a sequence of observations taken in a sequential time pattern.^24^ The significance of evaluating the effect of intervention through appropriate statistic modelling is progressively established and time series modelling implementing ITSA has been widely used in healthcare settings.

The intervention analysis is based on defined waves of the COVID-19 pandemic. The distribution of COVID-19 pandemic waves in England has varied over time, with different regions experiencing different levels of transmission and impact, however, in general, the waves have affected the entire country to some degree. The intervention model was extended to evaluate the effect of waves 1–3 of the COVID-19 pandemic. Three COVID-19 waves were identified in England: wave 1 from January to June 2020, wave 2 from June 2020 to April 2021 and wave 3 from April 2021 onwards.^25^

The main aim of this study was to conduct an ITSA to evaluate the impact of COVID-19 on antimicrobial consumption at the secondary care NHS Trust in England from April 2017 to September 2022. The secondary aim was to determine the trend of the other therapeutic agents for treating COVID-19 corticosteroids, antivirals, and monoclonal antibodies in a secondary care NHS Trust in England from April 2017 to September 2022.

## Methods

### Study design and setting

This retrospective ITSA was performed at the Mid Yorkshire Teaching NHS Trust, comprising three secondary care NHS hospitals: Pinderfields Hospital (750 beds), Dewsbury District Hospital (200 beds), Pontefract General Infirmary (50 beds) and community services in West Yorkshire, England. The Trust cares for 500 000 people, providing medical and surgical services, with one 30-bed intensive care unit (ICU), Haematology/Oncology, regional burns, regional spinal injuries and ambulatory care facilities. The study was approved by the University of Huddersfield Research Ethics Committee (SAS-SRIEC-11.1.22-2).

#### Data collection

Data were collected with the support of the pharmacy data analyst, using clinical systems including JAC (pharmacy software), Medchart (e-prescribing) and PPM+ (clinical notes from the hospital pharmacy information systems), and monthly antimicrobial consumption quantities from April 2017 to September 2022 were collected after applying inclusion and exclusion criteria. The antimicrobial consumption and other therapeutic agents for treating COVID-19 were gathered and converted into DDDs defined by WHO/Anatomical Therapeutic Chemical Classification 2022 index^26^ for systemic use only as ‘the assumed average maintenance dose per day for a drug used for its main indication in adults’.^27^ The WHO AWaRe classification was used.^28^ The gathered information was converted into DDDs per 1000 OBDs monthly.

#### Inclusion and exclusion criteria

All data were collected at the population level for the entire trust every month for hospitalized adult inpatients. Paediatric patients were excluded as DDDs defined by the WHO were intended for the adult population only. No paediatric patients, day cases, A&E or discharge prescriptions were included in the study.

### Analysis: intervention methodology

The intervention methodology used the Scientific Computing Associates software, which allows the integration of effective time series analysis and forecasting capacities.^29^ The collected data were equally spaced in the monthly time series. Data availability for antimicrobial and other therapeutic agents for COVID-19 treatments was from April 2017 to September 2022 with 66 monthly observations.

In this ITSA,^30^ we set out to evaluate the impact of COVID-19 on antimicrobial use and other treatments (remdesivir, steroids and monoclonal antibodies) through intervention analysis, as introduced by Box and Tiao in 1975.^31^ In the Box–Tiao intervention approach, a time series is represented by two distinct components: an underlying disturbance process, and the set of interventions in the series.^24^ The general form of the intervention model applied in this study is.


$$Y_{t}=C+\omega_{1}(B)I_{1t}+\omega_{2}(B)I_{2t}+\ldots +\omega_{m}(B)I_{mt}+N_{t}$$

where $I_{t}$ are binary indicators (0/1) that define the intervention periods. The term ω(B) is the effect(s) of the intervention concerning the base period. The term $N_{t}$ is called the disturbance and follows an autoregressive-moving-average (ARMA) process.

To account for a transition period, we compared antimicrobial consumption in the pre-intervention period to the overall COVID-19 intervention period, but we separately analysed the monthly effect in February–March 2020 from the monthly effect in the remaining intervention period, i.e. from April 2020 to September 2022. The structure of the intervention model is.


$$Y_{t}=C+\omega_{1}(B)I_{1t}+\omega_{2}(B)I_{2t}+N_{t}$$

where $I_{t}$ is a step function defined as


$$\mathrm{All}\;\mathrm{waves}(a)\equiv I_{1t}=\{\begin{array}{l} 1,\;t=\mathrm{February}\;\;\mathrm{to}\;\;\mathrm{March}\;\;2020\\ 0,\;\;\;\;\mathrm{otherwise} \end{array}$$

$$\mathrm{All}\;\mathrm{waves}(b)\equiv I_{2t}=\{\begin{array}{l} 1,\;\;\;\;\;t\geq \mathrm{April}\;2020\\ 0,\;\;\;\;\mathrm{otherwise}\;\;\;\;\;\;\;\;\; \end{array}$$

The disturbances, $N_{t}$, were identified for each antimicrobials series to determine the ARMA parameters needed to induce stationarity and remove serial correlation in the residuals, thus, rendering this a white noise process.

After analysing the overall COVID-19 intervention period, we applied intervention analysis based on the defined three waves of the COVID-19 pandemic in England. These were compared to the pre-intervention period. The February–March 2020 transition was introduced into the model that can be contrasted to the remaining months in wave 1.


$$Y_{t}=C+\omega_{1}(B)I_{1t}+\omega_{2}(B)I_{2t}+\;\omega_{3}(B)I_{3t}+\omega_{4}(B)I_{4t}+N_{t}$$

where,


$$\mathrm{Wave}\;1(a)\equiv I_{1t}=\{\begin{array}{l} 1,\;\;\;\;\;\;\;\;\;\;t=\mathrm{February}\;\;\mathrm{to}\;\;\mathrm{March}\;\;2020\\ 0,\;\;\;\;\;\;\;\;\;\;\;\;\;\;\;\;\;\;\;\;\;\;\;\;\;\;\;\;\;\;\;\;\;\;\;\;\mathrm{otherwise}\;\;\;\;\;\;\;\;\; \end{array}$$

$$\mathrm{Wave}\;1(b)\equiv I_{2t}=\{\begin{array}{l} 1,\;\;\;\;\;\;\;\;\;\;\;t=\mathrm{April}\;\;2020\;\;\mathrm{to}\;\;\mathrm{June}\;\;2020\\ 0,\;\;\;\;\;\;\;\;\;\;\;\;\;\;\;\;\;\;\;\;\;\;\;\;\;\;\;\;\;\;\;\;\;\;\;\;\;\;\;\;\;\;\mathrm{otherwise}\;\;\;\;\;\;\;\;\; \end{array}$$

$$\mathrm{Wave}\;2\equiv I_{3t}=\{\begin{array}{l} 1,\;\;\;\;\;\;\;\;\;\;\;t=\mathrm{July}\;\;2020\;\;\mathrm{to}\;\;\mathrm{April}\;\;2021\\ 0,\;\;\;\;\;\;\;\;\;\;\;\;\;\;\;\;\;\;\;\;\;\;\;\;\;\;\;\;\;\;\;\;\;\;\;\;\;\;\;\;\;\mathrm{otherwise}\;\;\;\;\;\;\;\;\; \end{array}$$

$$\mathrm{Wave}\;3\equiv I_{4t}=\{\begin{array}{l} 1,\;\;\;\;\;\;\;\;\;\;\;t=\mathrm{May}\;\;2021\;\;\mathrm{to}\;\;\mathrm{September}\;\;2022\\ 0,\;\;\;\;\;\;\;\;\;\;\;\;\;\;\;\;\;\;\;\;\;\;\;\;\;\;\;\;\;\;\;\;\;\;\;\;\;\;\;\;\;\;\;\;\;\;\;\;\;\;\;\;\mathrm{otherwise}\;\;\;\;\;\;\;\;\; \end{array}$$

The use of antibiotics was determined by applying ITSA. According to the obtained methods for conducting this study, three periods were defined; the first pre-intervention period (pre-pandemic period) from 2017 to January 2020 and transition periods were introduced as February 2020 to March 2020 and then the intervention period (pandemic period) from April 2020 to September 2022.

## Results

### Antibiotic consumption in all waves

Overall antibiotic consumption was not significantly changed from 826.4 DDDs per 1000 OBDs in the pre-interventional period (pre-pandemic period/base period) compared with 728.2 DDDs per 1000 OBDs (*P* = 0.0067; Table 1) in the intervention period (pandemic period) and antibiotic trends varied during the study period as shown in Figure S1 (available as Supplementary data at *JAC-AMR* Online). Increased consumption of combinations of penicillins, incl. B-lactamase inhibitors (J01CR; *P* = 0.2231), sulfamethoxazole and trimethoprim (*P* = 0.1817) were observed. Likewise, a decrease in consumption of various antibiotic classes was observed, including macrolides (*P* = 0.9968), fluoroquinolones (*P* = 0.0010) and glycopeptides (*P* = 0.0002), tetracyclines (*P* = 0.2754) and aminoglycosides (*P* = 0.0112). Few antibiotics showed statistical significance including first-generation cephalosporin (*P* < 0.0001) consumption was increased while second-generation cephalosporin (*P* < 0.0001) and imidazole derivates (*P* < 0.0001) showed decreased consumption trends during the pandemic period.

**Table 1.** Consumption of antibiotics and antifungals, corticosteroids and monoclonal antibodies during the study period April 2017 to September 2022 pre- and post-pandemic period

Antibiotic (J01)	Pre-pandemic period (April 2017 to Jan 2020) ^a^Base				Transition period (February–March 2020)				Pandemic period) (April 2020–September 2022) ^b^Interventional period				Noise model
	Constant	*P* value	Mean	SD	Coefficient (95% CI)	*P* value	Mean	SD	Coefficient (95% CI)	*P* value	Mean	SD	
Tetracyclines (J01A)	62.4	<0.0001	59.1	16.2	−7.44 (−23.10 to 8.21)	0.3507	75.1	6.7	−7.80 (−21.76 to 6.15)	0.2754	53.9	8.6	ARMA(1,0)
Amphenicols (J01B)	0.2	<0.0001	0.2	0.2	−0.18 (−0.49 to 0.12)	0.2306	0.0	0.0	−0.05 (−0.15 to 0.05)	0.3210	0.1	0.2	ARMA(0,0)
Penicillins with extended spectrum (J01CA)	63.0	<0.0001	62.8	8.9	−2.29 (−17.19 to 12.60)	0.7651	72.4	2.5	−5.75 (−13.77 to 2.27)	0.1633	56.1	11.2	ARMA(1,0)
B-lactam sensitive (J01CE)	86.6	<0.0001	87.1	10.9	2.57 (−15.23 to 20.39)	0.7727	88.6	8.6	−2.8703 (−9.02 to 3.27)	0.3610	83.9	12.8	ARMA(1,1)
B-lactam resistant (J01CF)	11.8	<0.0001	11.9	2.8	1.99 (−2.25 to 6.24)	0.3610	14.5	0.1	−2.93 (−5.16 to −0.70)	0.0115	8.8	3.0	ARMA(1,0)
Combinations of penicillins, incl. B-lactamase inhibitors (J01CR)	187.6	<0.0001	188.4	13.5	−23.27 (−53.25 to 6.71)	0.1308	186.4	24.1	7.91 (−4.80 to 20.63)	0.2231	193.7	23.5	ARMA(0,1)
First-generation cephalosporin (J01DB)	2.4	<0.0001	2.4	0.7	2.44 (1.27 to 3.61)	0.0001	4.6	1.9	1.51 (1.20 to 1.81)	<0.0001	3.9	0.8	ARMA(1,1)
Second-generation cephalosporin (J01DC)	4.6	<0.0001	4.6	1.1	−0.4161 (−1.87 to 1.04)	0.5774	4.1	0.5	−1.32 (−1.82 to −0.81)	<0.0001	3.2	1.0	ARMA(0,0)
Third-generation cephalosporin (J01DD)	16.6	<0.0001	16.2	4.5	1.47 (−3.49 to 6.4490)	0.5572	15.2	3.0	−4.70 (−7.32 to −2.07)	0.0008	11.8	1.9	ARMA(1,0)
Monobactams (J01DF)	3.9	<0.0001	3.7	2.2	−0.11 (−2.30 to 2.08)	0.9207	2.7	1.7	−1.46 (−3.29 to 0.36)	0.1190	2.7	1.1	ARMA(1,0)
Carbapenems (J01DH)	12.2	<0.0001	13.6	3.6	−1.79 (−6.37 to 2.77)	0.4441	13.0	1.3	−0.93 (−3.79 to 1.92)	0.5244	11.2	3.1	ARMA(1,1)
Other cephalosporin and penems (J01DI)	0.1	0.0265	0.1	0.2	−0.08 (−0.42 to 0.24)	0.5979	0.0	0.0	−0.03 (−0.15 to 0.07)	0.4989	0.1	0.3	ARMA(0,0)
Sulphonamide and trimethoprim (J01E)	31.3	<0.0001	31.9	5.2	6.87 (−0.81 to 14.56)	0.0831	37.2	5.2	2.33 (−1.07 to 5.75)	0.1817	33.9	5.1	ARMA(1,1)
Macrolides (J01FA)	96.0	<0.0001	101.7	24.9	−60.89 (−94.98 to −26.80)	0.0008	100.1	35.2	0.06 (−27.47 to 27.60)	0.9968	86.9	27.8	ARMA(1,1)
Lincosamides/clindamycin (J01FF)	13.3	<0.0001	13.4	5.1	−2.44 (−8.53 to 3.63)	0.4324	9.3	0.8	−4.40 (−7.29 to −1.52)	0.0037	8.8	2.2	ARMA(1,0)
Aminoglycosides (J01GB)	40.2	<0.0001	39.6	7.9	−7.48 (−17.89 to 2.91)	0.1604	34.6	3.9	−11.21 (−19.68 to −2.75)	0.0112	28.9	9.2	ARMA(1,0)
Fluoroquinolones (J01MA)	62.6	<0.0001	63.4	9.3	−15.46 (−31.79 to 0.87)	0.0673	55.8	11.9	−11.88 (−18.68 to −5.08)	0.0010	50.0	12.2	ARMA(0,1)
Glycopeptide (J01XA)	75.0	<0.0001	74.5	9.9	−15.59 (−30.54 to −0.64)	0.0444	78.3	5.7	−22.70 (−33.97 to −11.44)	0.0002	50.3	12.6	ARMA(1,0)
Polymyxins (J01XB)	0.1	0.0006	0.1	0.2	−0.06 (−0.30 to 0.17)	0.6048	0.0	0.0	−0.03 (−0.14 to 0.08)	0.5979	0.1	0.1	ARMA(1,0)
Steroid antibacterial (J01XC)	0.1	0.1604	0.3	0.6	0.62 (−0.18 to 1.43)	0.1359	0.3	0.4	0.24 (0.07 to 0.40)	0.0045	0.4	0.8	ARMA(1,1)
Imidazole derivative (J01XD)	31.4	<0.0001	30.7	5.4	−7.36 (−13.90 to −0.82)	0.0299	23.7	1.7	−12.20 (−16.41 to −7.99)	<0.0001	19.0	6.3	ARMA(1,2)
Nitrofuran derivative (J01XE)	17.5	<0.0001	17.1	4.0	1.76 (−3.30 to 6.84)	0.4927	17.8	2.1	0.16 (−2.09 to 2.42)	0.8891	17.7	3.0	ARMA(1,0)
Other antibacterial (J01XX)	3.1	0.0106	3.7	3.0	0.91 (−2.21 to 4.03)	0.5706	2.8	1.5	−0.11 (−3.00 to 2.78)	0.9365	3.0	1.7	ARMA(1,1)
**Total antibiotic**	**829**.**8**	**<0**.**0001**	**826**.**4**	**53**.**0**	**−108.79 (−216.00 to −1.59)**	**0**.**0497**	**836**.**8**	**102**.**4**	**−92.78 (−158.05 to −27.51)**	0.0067	**728**.**2**	**91**.**6**	**ARMA(1,0)**
**Antifungal (J02)**													
Amphotericin preparation (J02AA)	8.3	<0.0001	8.3	6.2	0.09 (−8.87 to 9.05)	0.9841	10.5	2.3	0.48 (−3.34 to 4.30)	0.8034	8.6	5.5	ARMA(1,0)
Triazole and tetrazole derivatives (J02AC)	9.0	0.0229	12.9	2.9	1.03 (−3.03 to 5.10)	0.6188	10.7	1.7	3.47 (−0.49 to 7.43)	0.0884	12.5	2.6	ARMA(1,1)
Other antimycotics (J02AX)	1.8	<0.0001	1.7	1.0	−0.33 (−1.97 to 1.30)	0.6905	1.7	0.9	0.0006 (−0.84 to 0.84)	0.9992	1.8	1.2	ARMA(1,0)
**Total antifungals**	**22**.**8**	**<0**.**0001**	**22**.**9**	**7**.**0**	**−2.59 (−12.49 to 7.30)**	**0**.**6048**	**22**.**9**	**0**.**3**	**0.34 (−4.14 to 4.84)**	**0**.**8812**	**22**.**9**	**6**.**0**	**ARMA(1,0)**
**Other therapeutic options for COVID-19 treatments**													
**Corticosteroids (H02)**													
Dexamethasone (H02AB02)	555.5	<0.0001	568.0	42.8	−61.00 (−215.12 to 93.10)	0.4382	546.5	7.2	−49.44 (−161.27 to 62.38)	0.3875	497.2	163.1	ARMA(1,0)
Hydrocortisone (H02AB09)	99.0	<0.0001	82.3	16.2	16.92 (−9.54 to 43.39)	0.2122	113.0	6.5	1.74 (−22.12 to 25.61)	0.8891	102.9	19.9	ARMA(1,1)
Prednisolone (H02AB06)	316.4	<0.0001	314.4	39.9	−27.60 (−81.43 to 26.21)	0.3162	321.1	32.8	−97.51 (−128.09 to −66.92)	<0.0001	218.7	33.1	ARMA(1,0)
**Total corticosteroids**	**960**.**4**	**<0**.**0001**	**964**.**7**	**59**.**7**	**−60.31 (−223.61 to 102.98)**	**0**.**4680**	**980**.**5**	**46**.**5**	**−138.62 (−250.06 to −27.19)**	**0**.**0170**	**818**.**8**	**164**.**0**	**ARMA(1,0)**
**Antivirals (J05)**													
Remdesivir (J05AB16)	0.72	0.0010	0.00	0.00	−0.42 (−1.44 to 0.5997)	0.415	0.00	0.00	−0.39 (−0.98 to 0.18)	0.1817	3.99	6.74	ARMA(1,0)
**Monoclonal antibodies (mABs)**													
Tocilizumab^c^ (L04AC07)	23.5	<0.0001	24.3	5.2	−3.72 (−21.03 to 13.58)	0.6759	16.1	2.9	4.14 (−8.31 to 16.60)	0.5116	28.1	18.2	ARMA(1,0)
Sarilumab^c^ (L04AC14)	0.0	0.001	0.0	0.0	0.00 (−10.33 to 10.33)	0.0009	0.0	0.0	4.08 (0.52 to 7.63)	0.0271	4.1	10.9	ARMA(0,0)
Baricitinib^c^ (L04AA37)	0.0	0.001	0.0	0.0	0.00 (−0.50 to 0.50)	0.0009	0.0	0.0	0.29 (0.1266 to 0.47)	0.0011	0.3	0.5	ARMA(0,0)
Sotrovimab^c^ (J06BD05)	0.0	0.001	0.0	0.0	0.00 (−0.03 to 0.03)	0.0009	0.0	0.0	0.01 (0.00 to 0.0248)	0.0292	0.0	0.0	ARMA(0,0)

^a^Base period (pre-pandemic period).

^b^Intervention period (pandemic period).

^c^Tocilizumab and Sarilumab are classified as interleukin (IL) inhibitors, Baricitinib is used as repurpose drugs for COVID patients and Sotrovimab is classified as antiviral monoclonal antibodies.

**Figure 1.**
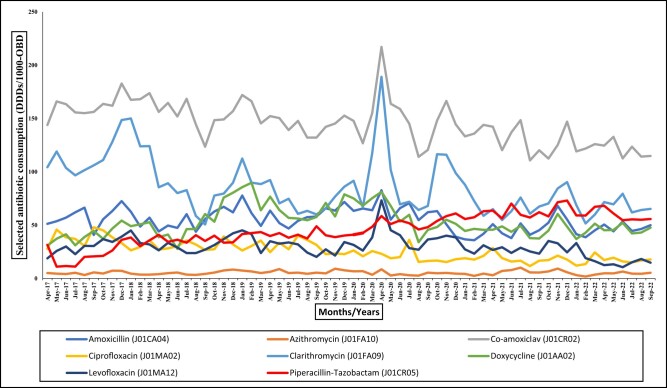
Consumption trends of selected antibiotics prescribed for RTIs during the study period from April 2017 to September 2022. These antibiotics were also prescribed for other indications, however, during the pandemic period they were mostly prescribed for RTIs.

Antibiotics commonly used for respiratory tract infections (RTI, Table 1, Figure 1) were also analysed individually. Consumption of piperacillin/tazobactam statistically significantly changed, increasing from 34.1 to 58.9 DDDs per 1000 OBDs (*P*≤0.0001) and ciprofloxacin significantly decreased from 32.2 to 18.5 DDDs per 1000 OBDs (*P* < 0.0001). Levofloxacin, azithromycin, doxycycline and amoxicillin showed a slight decrease in consumption; clarithromycin showed a decrease from 92.5 to 78.0 DDDs per 1000 OBDs (*P* = 0.9444) but did not reach statistical significance (Table 2). According to the WHO AWaRe classification, there were no statistically significant changes in the percentage consumption of Access (*P* = 0.6328), Watch (*P* = 0.7576) and Reserve (*P* = 0.9603) antibiotics (Table 3, Figure S2) during the study period.

**Table 2.** Consumption trends of selected individual antibiotics prescribed for RTI during the study period April 2017 to September 2022

RTI antibiotics,^c^ all waves	Pre-pandemic period (April 2017 to Jan 2020)^a^				Transition period (February–March 2020)				Pandemic period) (April 2020–September 2022)^b^				Noise model
	Constant	*P* value	Mean	SD	Coefficient (95% CI)	*P* value	Mean	SD	Coefficient (95% CI)	*P* value	Mean	SD	
Clarithromycin (J01FA09)	87.2	<0.0001	92.5	24.6	−56.31 (−88.32 to −24.29)	0.001	92.1	35.9	−0.88 (−27.75 to 25.98)	0.9444	78.0	26.6	ARMA(1,1)
Levofloxacin (J01MA12)	28.5	<0.0001	31.0	6.8	−16.29 (−29.14 to −3.43)	0.015	32.3	8.8	3.70 (−8.29 to 15.70)	0.5440	28.4	12.5	ARMA(1,0)
Ciprofloxacin (J01MA02)	32.4	<0.0001	32.2	6.6	−8.85 (−17.73 to 0.02)	0.054	23.3	3.4	−13.82 (−17.48 to −10.16)	<0.0001	18.5	4.8	ARMA(1,0)
Piperacillin-tazobactam (J01CR05)	35.9	<0.0001	34.1	9.7	6.81 (−3.39 to 17.01)	0.195	45.5	4.1	21.45 (12.16 to 30.74)	<0.0001	58.9	6.5	ARMA(1,0)
Co-amoxiclav (J01CR02)	152.8	<0.0001	154.3	13.0	−41.36 (−67.12 to −15.59)	0.002	140.9	20.0	−14.66 (−32.21 to 2.87)	0.1038	134.8	22.2	ARMA(1,0)
Azithromycin (J01FA10)	5.5	<0.0001	5.5	1.6	−1.54 (−4.34 to 1.25)	0.280	5.1	2.3	−0.31 (−1.65 to 1.02)	0.6471	5.0	2.0	ARMA(1,0)
Doxycycline (J01AA02)	57.7	<0.0001	55.1	16.5	−7.86 (−23.89 to 8.16)	0.336	71.7	5.0	−7.61 (−22.18 to 6.95)	0.3068	48.6	9.5	ARMA(1,0)
Amoxicillin (J01CA04)	58.0	<0.0001	57.9	8.7	−4.21 (−19.07 to 10.65)	0.577	64.7	1.1	−6.39 (−14.21 to 1.43)	0.1122	50.6	11.2	ARMA(1,0)

^a^Base period (pre-pandemic period).

^b^Intervention period (pandemic period).

^c^These antibiotics were also prescribed in other clinical indications.

**Table 3.** Consumption of Antibiotics according to WHO AWaRe Classification during the study period April 2017 to September 2022

ABx AWaRE classification	Pre-pandemic period (April 2017 to Jan 2020) ^a^Base				Transition period (February–March 2020)				Pandemic period) (April 2020–September 2022) ^b^Interventional period				Noise model
	Constant	*P* value	Mean	SD	Coefficient (95% CI)	*P* value	Mean	SD	Coefficient (95% CI)	*P* value	Mean	SD	
Access %	0.6	<0.0001	0.6	0.0	0.0300 (−0.0007 to 0.0607)	0.0592	0.6	0.0	−0.0051 (−0.0260 to 0.0158)	0.6328	0.6	0.0	ARMA(1,0)
Watch %	0.4	<0.0001	0.4	0.0	−0.0305 (−0.0616 to 0.0006)	0.0580	0.4	0.0	0.0030 (−0.0163 to 0.0223)	0.7576	0.4	0.0	ARMA(1,0)
Reserve %	0.0	0.001	0.0	0.0	0.0021 (−0.0034 to 0.0076)	0.4680	0.0	0.0	−0.0002 (−0.0067 to 0.0063)	0.9603	0.0	0.0	ARMA(1,1)

^a^Base period (pre-pandemic period).

^b^Intervention period (pandemic period). Abx, antibiotics.

### Antibiotic consumption in individual waves of the COVID-19 pandemic

Antibiotic consumption during the individual wave revealed maximum consumption in wave 1 920.3 DDDs per 1000 OBD (*P* = 0.0241) that declined to 684.8 DDDs per 1000 OBD (*P* < 0.0001) was shown as statistically significant. However, first-generation cephalosporin (*P* < 0.0001) showed more statistical significance in waves 2 and 3. Similar imidazole derivative (*P* < 0.0001) consumption was statistically significantly decreased in waves 2 and 3. Along with second-generation cephalosporin (*P* < 0.0001), aminoglycosides (*P* < 0.0001), fluoroquinolone (*P* < 0.0001), glycopeptide (*P* < 0.0001) and polymyxins (*P* < 0.0001), consumption was statistically significant decreased in wave 3. The consumption of several broad-spectrum antibiotics was raised, including variations in beta-lactam-sensitive and beta-lactam-resistant antibiotic consumption. Beta-lactam-sensitive antibiotics were determined as 80.1 DDDs per 1000 OBDs (*P* = 0.3115, wave 1), which increased to 87.2 DDDs per 1000 OBDs in wave 2 (*P* = 0.9921) and decreased to 82.6 DDDs per 1000 OBDs in wave 3 (*P* = 0.1848). Maximum consumption of beta-lactam-resistant antibiotics was 14.4 DDDs per 1000 OBDs (wave 1 compared with the pre-pandemic period); this decreased to 7.4 DDDs per 1000 OBDs in wave 2 (*P* = 0.0008) and marginally increased to 8.6 in wave 3 (*P* = 0.0035).

Individual antibiotic consumption tracked for RTI were clarithromycin, levofloxacin, piperacillin/tazobactam, co-amoxiclav, azithromycin, doxycycline and amoxicillin. Piperacillin/tazobactam consumption increased through the pandemic period from 54.6 (*P* = 0.0015, wave 1) to 55.9 (*P* = 0.0002, wave 2) to a maximum of 61.4 DDDs per 1000 OBDs (*P* = 0.0002, wave 3). We observed a statistically significant drop of ciprofloxacin (*P* < 0.0001) and co-amoxiclav (*P* < 0.0001) in wave 3. However, excluding ciprofloxacin and doxycycline, other RTI antibiotics consumption was raised in wave 1, azithromycin consumption was raised (*P* = 0.7880, wave 1) and the maximum reported in wave 3 (*P* = 0.2306) did not reach statistical significance.

### Antibiotic consumption according to WHO AWaRe classification in all wave and individual waves of the COVID-19 pandemic

According to WHO AWaRe classification, the percentage use of Watch antibiotics rose from 38% for pre-pandemic to 40% (*P* = 0.3610) during wave 1, and the Access category decreased from 61% pre-pandemic to 59% during wave 1 (*P* = 0.6188; Figure S2). However, this is not statistically significant and followed the analogous trend of antibiotics as per AWaRe classification as pre-pandemic (or base period) (Supplementary Tables S1–S3).

### Antifungal consumption in all waves and individual waves of the COVID-19 pandemic

Overall antifungal consumption showed no change from the pre-interventional period to the pandemic period, 22.9 to 22.9 DDDs per 1000 OBDs (*P* = 0.8812, Table 1). Slight increases in amphotericin derivative (*P* = 0.9841) and triazole and tetrazole derivatives (*P* = 0.6188) were observed in all waves.

In all three waves, there was a non-significant decrease for amphotericin preparations from 10.7 (*P* = 0.4441; first wave) to 10.3 DDDs per 1000 OBDs (*P* = 0.4741; second wave) to 7.2 DDDs per 1000 OBDs (*P* = 0.6905; third wave). Triazole and tetrazole derivatives consumption changed from the first wave [13.8 DDDs per 1000 OBDs (*P* = 0.6258)], to the second wave [11.3 DDDs per 1000 OBDs (*P* = 0.2976)] and to the third wave [3.0 DDDs per 1000 OBDs (*P* = 0.8891)] (Supplementary Table S4).

### Consumption of drugs used as COVID-19 therapeutic options during the COVID-19 pandemic

Corticosteroids (dexamethasone, hydrocortisone and prednisolone) decreased from 964.7 DDDs per 1000 OBDs to 818.8 DDDs per 1000 OBDs (*P* = 0.0170; Table 1). The use of corticosteroids was highest in the second wave at 936.1 DDDs per 1000 OBDs (*P* = 0.5639), and dexamethasone consumption was also higher in the second wave at 624.3 DDDs per 1000 OBDs (*P* = 0.4441), although these results did not reach statistical significance. Remdesivir showed maximum consumption in the second wave of 6.8 DDDs per 1000 OBDs (*P*≤0.0001). Monoclonal antibodies (mABs) were also used as a new treatment option with tocilizumab being the most common mAB used. The highest consumption of tocilizumab was in the second wave, i.e. 35.0 DDDs per 1000 OBDs (*P* = 0.2544), and sarilumab was used in the third wave, i.e. 7.2 DDDs per 1000 OBDs (*P* = 0.0007) (Supplementary Tables S5–S7).

**Figure 2.**
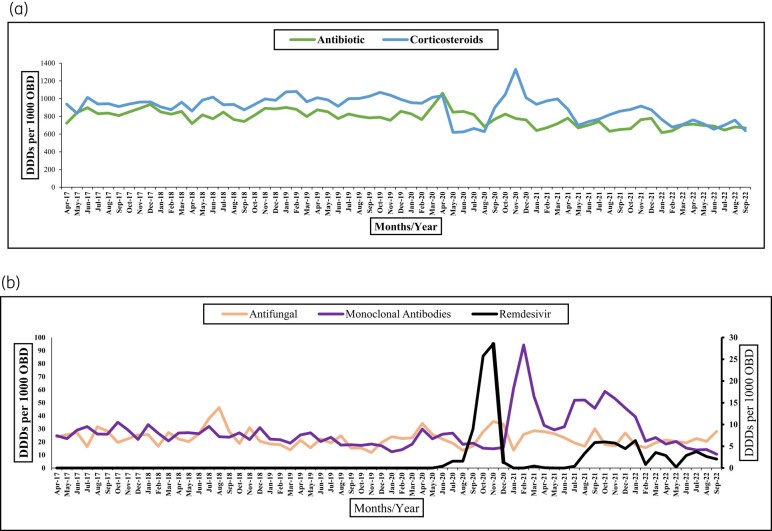
(a) Trends of antibiotics and corticosteroids during the study period from April 2017 to September 2022. On the *y*-axis: consumption of antibiotics (J01) and corticosteroids (H02)-for systematic use) and on the *x*-axis: months and years. (b) Trends of antifungal, monoclonal antibodies and remdesivir (antiviral) during the study period from April 2017 to September 2022. On the primary left *y*-axis are antifungal (J02) monoclonal antibodies mABs, the secondary right *y*-axis shows remdesivir (antiviral) and the *x*-axis is months and years.

With increased use of therapeutic options for COVID-19 treatment, antimicrobial consumption declined as shown in Figure 2(a) and (b) There may be an association of remdesivir and mABs with the decline in antimicrobial consumption after the first wave. However, this was not statistically significant for remdesivir (*P* = 0.605) and mABs (*P* = 0.341).

## Discussion

### Antibiotic consumption

The primary aim of this study was to determine antibiotic consumption trends during pandemic periods compared with the pre-pandemic period (base) at an acute NHS Trust. We introduced a transition period from February 2020 to March 2020, which was the peak of COVID-19.

Antibiotic treatments were extensively used with no evidence of bacterial co-infection.^13,16,32–34^ Various studies reported an increase in antibiotic prescriptions for patients with mild COVID-19 symptoms.^35–37^ Early studies measured peak antibiotic use during the first two waves.^38,39^ According to a recent report by the English Surveillance Programme for Antimicrobial Utilisation and Resistance, total antibiotic consumption declined by 5.3% between 2018 and 2020.^40^ In our study, there was no significant increase in antibiotic consumption during the overall pandemic period compared to the pre-pandemic period.^38^ Consistent with the findings of our study, a conducted study in Northern Ireland reported there was no difference in the total hospital antibiotic consumption between pre-COVID-19 and during the COVID-19 pandemic.^41^ Similar to our study time series, an analysis conducted in tertiary care hospitals in Italy showed no significant increase in antibiotic consumption during the pandemic period.^38^ Early studies suggested the highest antibiotic use was during the early phase of the pandemic.^38,39^ Consistent with the result of our study, a study conducted in Spain by Grau^42^ revealed increased overall antibiotic consumption during the first wave. However, the increased use of amoxicillin/clavulanate was also similar to studies conducted in Italy and Spain.^38,42^ Additionally, both studies also reported increased consumption of piperacillin/tazobactam, which was further evident in our study.

The increase in antibiotic consumption in the first wave was probably due to a lack of information, no available guidelines, no treatment options and suspected co-bacterial infections in patients with the SARS-CoV-2 virus.^19,42^ Our study showed a trend in antibiotic consumption in three waves in England and after introducing the NICE guideline in May 2020,^43^ highlighting the value of studying the pandemic impact on antimicrobial consumption per individual wave. A decline in antibiotic consumption was significantly observed in wave 3. The availability of other therapeutic options, social distancing, infection prevention and control and an intense global vaccination programme were also thought to be the contributing factors.

### Antibiotics used in respiratory tract infection (RTI)

The most frequently prescribed RTI antibiotics, including clarithromycin, levofloxacin, piperacillin/tazobactam, co-amoxiclav, doxycycline and amoxicillin showed a decreasing trend in wave 1. We observed increased consumption of piperacillin/tazobactam during the pandemic, with the largest increase in wave 3. However, this increased consumption is most probably a reflection of the prescriptions for other indications such as respiratory sepsis and abdominal infections prescribed for non-COVID-19 patients. The use of various combinations of antimicrobials (such as azithromycin and hydroxychloroquine) substantially increased during the first wave but decreased with the introduction of treatment guidelines.^5,6,44^

### Antibiotic consumption according to WHO AWaRe classification

The WHO AWaRe tool was developed to address inappropriate antibiotic use, antibiotic-related adverse events and drug costs.^45,46^ AWaRe classified antibiotics as Access; typically narrow spectrum e.g. amoxicillin, cefalexin, nitrofurantoin etc., Watch; broad-spectrum antibiotics such as fluoroquinolone, macrolide third-generation cephalosporin and Reserve; used as the last resort such as linezolid, meropenem, colistin.^47^ The cross-section study conducted by Mudenda *et al*. from Zambia represent data from 2022.^48^ This study concluded the Access group was dominant in comparison with the Watch and Reserved antibiotic groups. Also, the percentage use of the Access antibiotic category decreased and the Watch category marginally increased in wave 1. The Reserve category remained almost constant throughout the study period. However, overall, no significant changes were observed in comparison to the pre-pandemic and pandemic periods.

### Antifungal consumption

In 2022, the WHO report ‘Fungal priority pathogens list to guide research, development and public health action’^49^ highlighted the increased incidence of invasive fungal infections globally, particularly in the immunocompromised. A high incidence of mucormycosis in patients with COVID-19 was reported in India during the pandemic, particularly in wave 2, highlighting the need to limit irrational antifungal use.^50,51^ Overall antifungal consumption (J02) in our study period was consistent pre- and post-pandemic, whereas the use of antifungals in wave 1 was high. We demonstrated that amphotericin consumption declined in wave 3, while triazole and tetrazole derivatives decreased in wave 2 but increased from the pre-pandemic period in wave 3.

### Impact of other treatment options on antimicrobial consumption

The secondary aim of our study was to evaluate the other therapeutic options used to treat patients with COVID-19. Various clinical trials including RECOVERY and SOLIDARITY recruited patients globally and showed promising results for treatments such as dexamethasone, remdesivir and other monoclonal antibodies (mABs).^52,53^ The RECOVERY and ISARIC WHO trials found that dexamethasone reduced mortality among patients with severe COVID-19.^54,55^ Several antiviral drugs were repurposed for the treatment of COVID-19 including remdesivir, molnupiravir and tenofovir.^56^ Other drugs were part of clinical trials, such as hydroxychloroquine and lopinavir/ritonavir but were withheld from the guidelines due to lack of evidence for effectiveness.^57^ Remdesivir was effective in reducing hospitalization rates and mortality among patients with COVID-19 and was reserved for patients with severe disease who were hospitalized.^52,58–60^ The use of remdesivir at a trust level, according to local and national NHS trust guidelines updated in May 2020.^61^ Monoclonal antibodies were used in combination with other treatments, such as antiviral drugs or corticosteroids, to improve outcomes in patients with COVID-19. They work by binding to specific proteins on the surface of the SARS-CoV-2 virus, preventing the virus from entering and infecting healthy cells in the body.^62,63^ Tocilizumab was associated with a significant reduction of mechanical ventilatory support.^64–66^ These studies shifted the paradigm for COVID-19 treatment.

Our study shows that in comparison with the pre-pandemic period, there was no statistically significant difference in various antibiotic drug classes (J01) and antifungal (J02) consumption in the pandemic period in all waves. However, we observed a surge in antimicrobial consumption in the first wave, which declined due to promising outcomes of clinical trials and various other therapeutic agents such as corticosteroids, mABs and remdesivir were prescribed and improved patient outcomes. Further effective public health measures with massive vaccination programmes play a pivotal role in reducing SARs-CoV-2 virus transmission^67,68^ and may result in declining trends of antimicrobial consumption as well as other therapeutic agents such as corticosteroids, mABs or remdesivir.

#### Strength and limitation

The main strength of our study is that it covered an extended period of observation monthly for 66 months. In comparison with other published studies, our study mainly focused on primary care and data represented from secondary care were not collected every month. This helped us to deeply understand consumption, particularly during the pandemic. This approach enabled us to introduce a transition period from February to March 2020. It also determined the antimicrobial trends in three waves of the COVID-19 pandemic in England. Further, our study determined trends in the adoption of various therapeutic agents for COVID-19 treatment including the use of corticosteroids, antivirals and monoclonal antibodies used during the pandemic, which were initiated as repurposed medications to treat COVID-19. This study also presents data for each wave and determines the shift in consumption of antimicrobials and COVID-19 new treatments.

The study has some limitations. This study was conducted at the population level. Information about patients’ characteristics and known prevalence of indications pre-COVID-19 and throughout the study period was not available. The description of the patient case mix would have helped better the interpretation of the findings.^69,70^ However, this level of information was not available.

The study data were sourced from the pharmacy dispensing system by both location and consultant in charge, so it was not possible to accurately break down antimicrobial use to ICU and non-ICU.

Additionally, the study was undertaken at one Trust in England and would benefit from a multicentre study focused on hospitalized patients; discharged or outpatient services were excluded from the study. Further work to examine outpatient antimicrobials would provide more insights into antimicrobial use during the COVID-19 pandemic. One of the main reasons to exclude paediatric patients was the specialist tertiary paediatric referral centre near to the Trust location; almost all high-risk paediatric patients were cared for outside of the Trust so paediatric numbers were so small as to not allow for any meaningful conclusions in the study.

### Conclusion

This study determined the trends of antimicrobial consumption pre-pandemic and during the pandemic and antimicrobial consumption trends in different waves in England. The new guidelines for treating COVID-19 were introduced. The rapid, more advanced research and publications during the pandemic significantly affected the reduction of antimicrobial use. Furthermore, the fluctuating trends in the initial wave emphasize advocating antimicrobial stewardship activities, AMS implementation and preparedness for pandemics in the future.

## Supplementary Material

dlae013_Supplementary_Data

## Supplementary data

Figures S1 and S2 and Tables S1–S7 are available as Supplementary data at *JAC-AMR* Online.
