# Supplementary material for: Antimicrobial consumption in an acute NHS Trust during the COVID-19 pandemic: intervention time series analysis
Source: JAC Antimicrob Resist. 2024 Feb 7;6(1):dlae013. doi: 10.1093/jacamr/dlae013 (PMC10848649; doi:10.1093/jacamr/dlae013)
Supplement: dlae013_Supplementary_Data [file dlae013_supplementary_data.docx]

**Supplementary Material**

**Table S1: Antibiotic consumption during all waves of the COVID-19 pandemic period (February 2020 to September 2022).**

| **Transition Period (February 2020-March 2020)** | **Constant** | **p-value** | **Coefficient (95% CI)** | **p-value** | **Mean** | **SD** | **Noise Model** |
| --- | --- | --- | --- | --- | --- | --- | --- |
| Tetracyclines (J01A) | 62.6 | <0.0001 | -5.7664 (-22.5268 to 10.9940) | 0.4989 | 75.1 | 6.7 | ARMA(1,0) |
| Amphenicols (J01B) | 0.2 | <0.0001 | -0.1893 (-0.4941 to 0.1155) | 0.2268 | 0.0 | 0.0 | ARMA(0,0) |
| Penicillins with extended-spectrum (J01CA) | 63.2 | <0.0001 | 4.3369 (-9.7825 to 18.4563) | 0.5440 | 72.4 | 2.5 | ARMA(1,0) |
| B-lactam sensitive (J01CE) | 87.1 | <0.0001 | 1.5068 (-14.8166 to 17.8302) | 0.8577 | 88.6 | 8.6 | ARMA(0,0) |
| B-lactam resistant (J01CF) | 11.9 | <0.0001 | 3.5554 (-0.3529 to 7.4637) | 0.0781 | 14.5 | 0.1 | ARMA(1,0) |
| Combination of penicillins/B-lactam inhibitors (J01CR) | 189.1 | <0.0001 | -10.7855 (-35.6634 to 14.0924) | 0.3984 | 186.4 | 24.1 | ARMA(1,0) |
| 1^st^ Generation Cephalosporin (J01DB) | 2.4 | <0.0001 | 2.2335 (1.1086 to 3.3584) | 0.0002 | 4.6 | 1.9 | ARMA(0,0) |
| 2^nd^ Generation Cephalosporin (J01DC) | 4.6 | <0.0001 | -0.4161 (-1.8660 to 1.0338) | 0.5706 | 4.1 | 0.5 | ARMA(0,0) |
| 3^rd^ Generation Cephalosporin (J01DD) | 16.6 | <0.0001 | 2.0662 (-3.1332 to 7.2656) | 0.4382 | 15.2 | 3.0 | ARMA(1,0) |
| Monobactams (J01DF) | 3.8 | <0.0001 | -0.6342 (-2.9204 to 1.6520) | 0.5842 | 2.7 | 1.7 | ARMA(1,0) |
| Carbapenems (J01DH) | 13.4 | <0.0001 | -1.7514 (-6.4867 to 2.9839) | 0.4680 | 13.0 | 1.3 | ARMA(1,0) |
| Other cephalosporin and penems (J01DI) | 0.1 | 0.0234 | -0.0898 (-0.4133 to 0.2337) | 0.5842 | 0.0 | 0.0 | ARMA(0,0) |
| Sulphonamide and trimethoprim (J01E) | 31.9 | <0.0001 | 5.3155 (-1.6335 to 12.2645) | 0.1358 | 37.2 | 5.2 | ARMA(0,0) |
| Macrolides (J01FA) | 98.9 | <0.0001 | -35.2735 (-69.8547 to -0.6923) | 0.0486 | 100.1 | 35.2 | ARMA(1,0) |
| Lincosamides / Clindamycin (J01FF) | 13.3 | <0.0001 | -2.3678 (-8.5855 to 3.8499) | 0.4560 | 9.3 | 0.8 | ARMA(1,0) |
| Other Aminoglycosides (J01GB) | 40.6 | <0.0001 | -2.8849 (-13.0347 to 7.2649) | 0.5774 | 34.6 | 3.9 | ARMA(1,0) |
| Fluoroquinolones (J01MA) | 64.2 | <0.0001 | -12.2215 (-25.6541 to 1.2111) | 0.0781 | 55.8 | 11.9 | ARMA(1,0) |
| Glycopeptide (J01XA) | 75.6 | <0.0001 | -6.7023 (-21.2098 to 7.8052) | 0.3662 | 78.3 | 5.7 | ARMA(1,0) |
| Polymyxins (J01XB) | 0.1 | 0.0006 | -0.0811 (-0.3289 to 0.1667) | 0.5180 | 0.0 | 0.0 | ARMA(1,0) |
| Steroid antibacterial (J01XC) | 0.3 | 0.0090 | -0.0131 (-1.0014 to 0.9752) | 0.9762 | 0.3 | 0.4 | ARMA(0,0) |
| Imidazole derivative (J01XD) | 30.4 | <0.0001 | -4.4252 (-11.1606 to 2.3102) | 0.2016 | 23.7 | 1.7 | ARMA(2,0) |
| Nitrofuran derivative (J01XE) | 17.5 | <0.0001 | 1.6799 (-3.4575 to 6.8173) | 0.5244 | 17.8 | 2.1 | ARMA(1,0) |
| Other antibacterial (J01XX) | 3.5 | <0.0001 | 0.3980 (-3.0595 to 3.8555) | 0.8188 | 2.8 | 1.5 | ARMA(1,0) |
| **Total Antibiotic** | **830.5** | **<0.0001** | **-5.6183 (-88.1554 to 76.9188)** | **0.8970** | **836.80** | **102.35** | **ARMA(1,0)** |
| **WAVE 1 (April 2020- June 2020)** | | | | | | | |
| Tetracyclines (J01A) | 62.6 | <0.0001 | -4.5326 (-23.7657 to 14.7005) | 0.6471 | 70.1 | 11.5 | ARMA(1,0) |
| Amphenicols (J01B) | 0.2 | <0.0001 | 0.0459 (-0.2065 to 0.2983) | 0.7200 | 0.2 | 0.1 | ARMA(0,0) |
| Penicillins with extended-spectrum (J01CA) | 63.2 | <0.0001 | 8.7440 (-4.0553 to 21.5433) | 0.1817 | 73.7 | 12.6 | ARMA(1,0) |
| B-lactam sensitive (J01CE) | 87.1 | <0.0001 | -7.0054 (-20.5172 to 6.5064) | 0.3115 | 80.1 | 22.0 | ARMA(0,0) |
| B-lactam resistant (J01CF) | 11.9 | <0.0001 | 2.7777 (-0.9285 to 6.4839) | 0.1437 | 14.4 | 3.1 | ARMA(1,0) |
| Combination of penicillins/B-lactam inhibitors (J01CR) | 189.1 | <0.0001 | 44.5380 (21.4492 to 67.6268) | 0.0003 | 234.5 | 35.6 | ARMA(1,0) |
| 1^st^ Generation Cephalosporin (J01DB) | 2.4 | <0.0001 | 1.4308 (0.4996 to 2.3620) | 0.0035 | 3.8 | 1.2 | ARMA(0,0) |
| 2^nd^ Generation Cephalosporin (J01DC) | 4.6 | <0.0001 | -0.8505 (-2.0506 to 0.3496) | 0.1662 | 3.7 | 1.6 | ARMA(0,0) |
| 3^rd^ Generation Cephalosporin (J01DD) | 16.6 | <0.0001 | -2.9113 (-7.7467 to 1.9241) | 0.2384 | 13.8 | 2.5 | ARMA(1,0) |
| Monobactams (J01DF) | 3.8 | <0.0001 | -2.6766 (-5.1407 to -0.2125) | 0.0361 | 1.7 | 0.7 | ARMA(1,0) |
| Carbapenems (J01DH) | 13.4 | <0.0001 | 2.3638 (-1.8083 to 6.5359) | 0.2668 | 15.6 | 3.6 | ARMA(1,0) |
| Other cephalosporin and penems (J01DI) | 0.1 | 0.0234 | -0.0898 (-0.3577 to 0.1781) | 0.5116 | 0.0 | 0.0 | ARMA(0,0) |
| Sulphonamide and trimethoprim (J01E) | 31.9 | <0.0001 | -2.6482 (-8.4002 to 3.1038) | 0.3661 | 29.3 | 1.4 | ARMA(0,0) |
| Macrolides (J01FA) | 98.9 | <0.0001 | 23.9961 (-15.5054 to 63.4976) | 0.2345 | 129.4 | 67.5 | ARMA(1,0) |
| Lincosamides / Clindamycin (J01FF) | 13.3 | <0.0001 | -4.1291 (-9.8332 to 1.5750) | 0.1575 | 8.7 | 1.3 | ARMA(1,0) |
| Other Aminoglycosides (J01GB) | 40.6 | <0.0001 | 0.5086 (-9.8199 to 10.8371) | 0.9207 | 45.4 | 9.8 | ARMA(1,0) |
| Fluoroquinolones (J01MA) | 64.2 | <0.0001 | 7.2290 (-5.3010 to 19.7590) | 0.2585 | 73.7 | 19.7 | ARMA(1,0) |
| Glycopeptide (J01XA) | 75.6 | <0.0001 | -7.5675 (-21.9296 to 6.7946) | 0.3022 | 74.6 | 11.7 | ARMA(1,0) |
| Polymyxins (J01XB) | 0.1 | 0.0006 | -0.0995 (-0.3278 to 0.1288) | 0.3929 | 0.1 | 0.1 | ARMA(1,0) |
| Steroid antibacterial (J01XC) | 0.3 | 0.0090 | -0.0347 (-0.8528 to 0.7834) | 0.9365 | 0.3 | 0.5 | ARMA(0,0) |
| Imidazole derivative (J01XD) | 30.4 | <0.0001 | -1.3973 (-7.7021 to 4.9075) | 0.6614 | 29.3 | 1.9 | ARMA(0,2) |
| Nitrofuran derivative (J01XE) | 17.5 | <0.0001 | -0.0712 (-4.7643 to 4.6219) | 0.9762 | 16.1 | 2.0 | ARMA(1,0) |
| Other antibacterial (J01XX) | 3.5 | <0.0001 | -2.7487 (-6.3083 to 0.8109) | 0.1334 | 1.7 | 1.4 | ARMA(1,0) |
| **Total Antibiotic** | **830.5** | **<0.0001** | **84.7197 (12.4335 to 157.0059)** | **0.0241** | **920.3** | **121.0** | **ARMA(1,0)** |
| **WAVE 2 (July 2020 - April 2021)** | | | | | | | |
| Tetracyclines (J01A) | 62.6 | <0.0001 | -7.7083 (-24.0688 to 8.6522) | 0.3558 | 53.1 | 6.9 | ARMA(1,0) |
| Amphenicols (J01B) | 0.2 | <0.0001 | -0.1106 (-0.2613 to 0.0401) | 0.1518 | 0.1 | 0.1 | ARMA(0,0) |
| Penicillins with extended-spectrum (J01CA) | 63.2 | <0.0001 | -6.0235 (-14.5940 to 2.5470) | 0.1723 | 55.5 | 12.3 | ARMA(1,0) |
| B-lactam sensitive (J01CE) | 87.1 | <0.0001 | 0.0467 (-8.0238 to 8.1172) | 0.9921 | 87.2 | 13.8 | ARMA(0,0) |
| B-lactam resistant (J01CF) | 11.9 | <0.0001 | -4.5778 (-7.1420 to -2.0136) | 0.0008 | 7.4 | 1.9 | ARMA(1,0) |
| Combination of penicillins/B-lactam inhibitors (J01CR) | 189.1 | <0.0001 | 8.5252 (-6.4565 to 23.5069) | 0.2668 | 195.3 | 19.1 | ARMA(1,0) |
| 1^st^ Generation Cephalosporin (J01DB) | 2.4 | <0.0001 | 1.6138 (1.0577 to 2.1699) | <0.0001 | 4.0 | 0.7 | ARMA(0,0) |
| 2^nd^ Generation Cephalosporin (J01DC) | 4.6 | <0.0001 | -1.3077 (-2.0246 to -0.5908) | 0.0006 | 3.3 | 1.2 | ARMA(0,0) |
| 3^rd^ Generation Cephalosporin (J01DD) | 16.6 | <0.0001 | -4.8278 (-8.3257 to -1.3299) | 0.0084 | 11.5 | 1.3 | ARMA(1,0) |
| Monobactams (J01DF) | 3.8 | <0.0001 | -1.3385 (-3.4823 to 0.8053) | 0.2231 | 2.0 | 0.4 | ARMA(1,0) |
| Carbapenems (J01DH) | 13.4 | <0.0001 | -1.2781 (-4.0221 to 1.4659) | 0.3610 | 11.9 | 3.1 | ARMA(1,0) |
| Other cephalosporin and penems (J01DI) | 0.1 | 0.0234 | 0.0619 (-0.0981 to 0.2219) | 0.4500 | 0.2 | 0.5 | ARMA(0,0) |
| Sulphonamide and trimethoprim (J01E) | 31.9 | <0.0001 | 3.8274 (0.3917 to 7.2631) | 0.0321 | 35.8 | 5.8 | ARMA(0,0) |
| Macrolides (J01FA) | 98.9 | <0.0001 | -0.8779 (-32.9566 to 31.2008) | 0.9603 | 89.3 | 22.0 | ARMA(1,0) |
| Lincosamides / Clindamycin (J01FF) | 13.3 | <0.0001 | -4.1480 (-8.1044 to -0.1916) | 0.0424 | 9.0 | 2.1 | ARMA(1,0) |
| Other Aminoglycosides (J01GB) | 40.6 | <0.0001 | -8.8816 (-17.1138 to -0.6494) | 0.0370 | 32.2 | 9.1 | ARMA(1,0) |
| Fluoroquinolones (J01MA) | 64.2 | <0.0001 | -10.5876 (-18.7653 to -2.4099) | 0.0131 | 52.1 | 7.2 | ARMA(1,0) |
| Glycopeptide (J01XA) | 75.6 | <0.0001 | -19.6349 (-29.9727 to -9.2971) | 0.0004 | 52.4 | 13.4 | ARMA(1,0) |
| Polymyxins (J01XB) | 0.1 | 0.0006 | -0.0215 (-0.1775 to 0.1345) | 0.7880 | 0.1 | 0.1 | ARMA(1,0) |
| Steroid antibacterial (J01XC) | 0.3 | 0.0090 | 0.2265 (-0.2623 to 0.7153) | 0.3661 | 0.5 | 1.4 | ARMA(0,0) |
| Imidazole derivative (J01XD) | 30.4 | <0.0001 | -9.6014 (-13.7975 to -5.4053) | <0.0001 | 21.0 | 7.9 | ARMA(2,0) |
| Nitrofuran derivative (J01XE) | 17.5 | <0.0001 | 0.1293 (-2.9709 to 3.2295) | 0.9365 | 17.5 | 4.2 | ARMA(1,0) |
| Other antibacterial (J01XX) | 3.5 | <0.0001 | -0.2065 (-3.0924 to 2.6794) | 0.8891 | 3.1 | 0.9 | ARMA(1,0) |
| **Total Antibiotic** | **830.5** | **<0.0001** | **-82.5791 (-126.5840 to -38.5742)** | **0.0004** | **744.3** | **63.1** | **ARMA(1,0)** |
| **WAVE 3 (April 2021 - September 2022)** | | | | | | | |
| Tetracyclines (J01A) | 62.6 | <0.0001 | -9.4380 (-24.7258 to 5.8498) | 0.2269 | 51.5 | 5.9 | ARMA(1,0) |
| Amphenicols (J01B) | 0.2 | <0.0001 | -0.0378 (-0.1623 to 0.0867) | 0.5506 | 0.2 | 0.2 | ARMA(0,0) |
| Penicillins with extended-spectrum (J01CA) | 63.2 | <0.0001 | -9.8633 (-17.1330 to -2.5936) | 0.0096 | 53.4 | 7.4 | ARMA(1,0) |
| B-lactam sensitive (J01CE) | 87.1 | <0.0001 | -4.5385 (-11.2024 to 2.1254) | 0.1848 | 82.6 | 10.9 | ARMA(0,0) |
| B-lactam resistant (J01CF) | 11.9 | <0.0001 | -3.3968 (-5.6026 to -1.1910) | 0.0035 | 8.6 | 2.4 | ARMA(1,0) |
| Combination of penicillins/B-lactam inhibitors (J01CR) | 189.1 | <0.0001 | -4.6184 (-17.2782 to 8.0414) | 0.4741 | 185.5 | 15.9 | ARMA(1,0) |
| 1^st^ Generation Cephalosporin (J01DB) | 2.4 | <0.0001 | 1.5355 (1.0763 to 1.9947) | <0.0001 | 3.9 | 0.9 | ARMA(0,0) |
| 2^nd^ Generation Cephalosporin (J01DC) | 4.6 | <0.0001 | -1.4102 (-2.0022 to -0.8182) | <0.0001 | 3.2 | 0.8 | ARMA(0,0) |
| 3^rd^ Generation Cephalosporin (J01DD) | 16.6 | <0.0001 | -5.1089 (-8.1657 to -2.0521) | 0.0016 | 11.7 | 2.0 | ARMA(1,0) |
| Monobactams (J01DF) | 3.8 | <0.0001 | -0.8459 (-2.8659 to 1.1741) | 0.4152 | 3.3 | 1.1 | ARMA(1,0) |
| Carbapenems (J01DH) | 13.4 | <0.0001 | -3.4811 (-5.7864 to -1.1758) | 0.0042 | 9.9 | 2.2 | ARMA(1,0) |
| Other cephalosporin and penems (J01DI) | 0.1 | 0.0234 | -0.0898 (-0.2220 to 0.0424) | 0.1848 | 0.0 | 0.0 | ARMA(0,0) |
| Sulphonamide and trimethoprim (J01E) | 31.9 | <0.0001 | 1.6506 (-1.1862 to 4.4874) | 0.2543 | 33.6 | 4.6 | ARMA(0,0) |
| Macrolides (J01FA) | 98.9 | <0.0001 | -18.1412 (-48.4073 to 12.1249) | 0.2423 | 78.0 | 11.4 | ARMA(1,0) |
| Lincosamides / Clindamycin (J01FF) | 13.3 | <0.0001 | -4.6174 (-7.9889 to -1.2459) | 0.0088 | 8.8 | 2.5 | ARMA(1,0) |
| Aminoglycosides (J01GB) | 40.6 | <0.0001 | -16.4999 (-23.8129 to -9.1869) | <0.0001 | 24.0 | 3.5 | ARMA(1,0) |
| Fluoroquinolones (J01MA) | 64.2 | <0.0001 | -19.6444 (-26.5617 to -12.7271) | <0.0001 | 44.5 | 7.2 | ARMA(1,0) |
| Glycopeptide (J01XA) | 75.6 | <0.0001 | -30.7138 (-39.7602 to -21.6674) | <0.0001 | 44.8 | 5.2 | ARMA(1,0) |
| Polymyxins (J01XB) | 0.1 | 0.0006 | -0.0206 (-0.1538 to 0.1126) | 0.7576 | 0.1 | 0.1 | ARMA(1,0) |
| Steroid antibacterial (J01XC) | 0.3 | 0.0090 | -0.0576 (-0.4611 to 0.3459) | 0.7804 | 0.3 | 0.4 | ARMA(0,0) |
| Imidazole derivative (J01XD) | 30.4 | <0.0001 | -14.3240 (-17.8661 to -10.7819) | <0.0001 | 16.0 | 2.0 | ARMA(2,0) |
| Nitrofuran derivative (J01XE) | 17.5 | <0.0001 | 0.2386 (-2.4018 to 2.8790) | 0.8577 | 18.0 | 2.3 | ARMA(1,0) |
| Other antibacterial (J01XX) | 3.5 | <0.0001 | -0.4243 (-3.0529 to 2.2043) | 0.7500 | 3.1 | 2.0 | ARMA(1,0) |
| **Total Antibiotic** | **830.5** | **<0.0001** | **-146.4314 (-183.1126 to -109.7502)** | <0.0001 | **684.8** | **45.5** | **ARMA(1,0)** |

**Table S2: Selected individual antibiotic consumption prescribed for Respiratory Tract Infections (February 2020 to September 2022)**

| **Transition Period (February 2020-March 2020)** | **Constant** | **p-value** | **Coefficient (95% CI)** | **p-value** | **Mean** | **SD** | **Noise Model** |
| --- | --- | --- | --- | --- | --- | --- | --- |
| Amoxicillin (J01CA02) | 58.2 | <0.0001 | 2.2255 (-11.7083 to 16.1593) | 0.7576 | 64.7 | 1.1 | ARMA(1,0) |
| Amoxicillin/Clavulanic acid (J01CR02) | 154.8 | <0.0001 | -19.8535 (-41.1591 to 1.4521) | 0.0703 | 140.9 | 20.0 | ARMA(1,0) |
| Azithromycin (J01FA10) | 5.5 | <0.0001 | -0.4100 (-3.4403 to 2.6203) | 0.7880 | 5.1 | 2.3 | ARMA(0,0) |
| Ciprofloxacin (J01MA02) | 32.4 | <0.0001 | -8.8144 (-17.6227 to -0.0061) | 0.0531 | 23.3 | 3.4 | ARMA(1,0) |
| Clarithromycin (J01FA09) | 89.6 | <0.0001 | -32.7171 (-65.3974 to -0.0368) | 0.0531 | 92.1 | 35.9 | ARMA(1,0) |
| Doxycycline (J01AA02) | 58.0 | <0.0001 | -6.1954 (-23.3624 to 10.9716) | 0.4802 | 71.7 | 5.0 | ARMA(1,0) |
| Levofloxacin (J01MA12) | 31.8 | <0.0001 | -5.1176 (-15.6301 to 5.3949) | 0.3406 | 32.3 | 8.8 | ARMA(1,0) |
| Piperacillin/tazobactam (J01CR05) | 35.8 | <0.0001 | 6.3268 (-4.4125 to 17.0661) | 0.2503 | 45.5 | 4.1 | ARMA(1,0) |
| **WAVE 1 (April 2020- June 2020)** | | | | | | | |
| Amoxicillin (J01CA04) | 58.2 | <0.0001 | 8.2946 (-4.2933 to 20.8825) | 0.1982 | 67.9 | 13.9 | ARMA(1,0) |
| Amoxicillin/Clavulanic acid (J01CR02) | 154.8 | <0.0001 | 23.8519 (4.1844 to 43.5194) | 0.0198 | 179.9 | 32.3 | ARMA(1,0) |
| Azithromycin (J01FA10) | 5.5 | <0.0001 | 0.3487 (-2.1605 to 2.8579) | 0.7880 | 5.1 | 3.0 | ARMA(1,0) |
| Ciprofloxacin (J01MA02) | 32.4 | <0.0001 | -13.5971 (-21.3735 to -5.8207) | 0.0010 | 20.7 | 2.2 | ARMA(1,0) |
| Clarithromycin (J01FA09) | 89.6 | <0.0001 | 22.0623 (-15.7318 to 59.8564) | 0.2544 | 120.1 | 61.8 | ARMA(1,0) |
| Doxycycline (J01AA02) | 58.0 | <0.0001 | -4.4743 (-24.2838 to 15.3352) | 0.6614 | 67.5 | 13.9 | ARMA(1,0) |
| Levofloxacin (J01MA12) | 31.8 | <0.0001 | 20.6428 (9.2991 to 31.9865) | 0.0007 | 53.0 | 17.7 | ARMA(1,0) |
| Piperacillin/tazobactam (J01CR05) | 35.8 | <0.0001 | 20.3449 (8.2515 to 32.4383) | 0.0015 | 54.6 | 3.8 | ARMA(1,0) |
| **WAVE 2 (July 2020 - April 2021)** | | | | | | | |
| Amoxicillin (J01CA04) | 58.2 | <0.0001 | -6.0693 (-14.4381 to 2.2995) | 0.1575 | 50.6 | 12.2 | ARMA(1,0) |
| Amoxicillin/Clavulanic acid (J01CR02) | 154.8 | <0.0001 | -13.5736 (-26.2959 to -0.8513) | 0.0396 | 139.4 | 14.7 | ARMA(1,0) |
| Azithromycin (J01FA10) | 5.5 | <0.0001 | -1.6459 (-3.3736 to 0.0818) | 0.0646 | 4.0 | 1.1 | ARMA(1,0) |
| Ciprofloxacin (J01MA02) | 32.4 | <0.0001 | -10.9388 (-16.0462 to -5.8314) | 0.0001 | 20.5 | 6.8 | ARMA(1,0) |
| Clarithromycin (J01FA09) | 89.6 | <0.0001 | 1.2065 (-30.3880 to 32.8010) | 0.9365 | 82.0 | 21.6 | ARMA(1,0) |
| Doxycycline (J01AA02) | 58.0 | <0.0001 | -7.3263 (-24.2728 to 9.6202) | 0.3984 | 47.6 | 6.8 | ARMA(1,0) |
| Levofloxacin (J01MA12) | 31.8 | <0.0001 | -0.4214 (-8.3449 to 7.5021) | 0.9207 | 31.7 | 6.0 | ARMA(1,0) |
| Piperacillin/tazobactam (J01CR05) | 35.8 | <0.0001 | 22.2574 (11.3121 to 33.2027) | 0.0002 | 55.9 | 6.0 | ARMA(1,0) |
| **WAVE 3 (April 2021 - September 2022)** | | | | | | | |
| Amoxicillin (J01CA04) | 58.2 | <0.0001 | -10.7675 (-17.8504 to -3.6846) | 0.0039 | 47.5 | 7.4 | ARMA(1,0) |
| Amoxicillin/Clavulanic acid (J01CR02) | 154.8 | <0.0001 | -31.0285 (-41.7479 to -20.3091) | 0.0000 | 124.2 | 11.3 | ARMA(1,0) |
| Azithromycin (J01FA10) | 5.5 | <0.0001 | 0.7623 (-0.4749 to 1.9995) | 0.2306 | 6.3 | 1.9 | ARMA(0,0) |
| Ciprofloxacin (J01MA02) | 32.4 | <0.0001 | -15.5296 (-19.8130 to -11.2462) | 0.0000 | 17.0 | 3.1 | ARMA(1,0) |
| Clarithromycin (J01FA09) | 89.6 | <0.0001 | -18.6076 (-48.8508 to 11.6356) | 0.2307 | 68.2 | 10.1 | ARMA(1,0) |
| Doxycycline (J01AA02) | 58.0 | <0.0001 | -9.6580 (-25.6668 to 6.3508) | 0.2384 | 45.8 | 5.9 | ARMA(1,0) |
| Levofloxacin (J01MA12) | 31.8 | <0.0001 | -4.1337 (-10.2159 to 1.9485) | 0.1849 | 27.2 | 5.9 | ARMA(1,0) |
| Piperacillin/tazobactam (J01CR05) | 35.8 | <0.0001 | 21.7324 (10.7613 to 32.7035) | 0.0002 | 61.4 | 6.2 | ARMA(1,0) |

^a^ These Antibiotics may be used for other clinical indications as well.

**Table S3: Antifungal consumption during all waves of the COVID-19 pandemic period (February 2020 to September 2022)**

| **Transition Period (February 2020-March 2020)** | **Constant** | **p-value** | **Coefficient (95% CI)** | **p-value** | **Mean** | **SD** | **Noise Model** |
| --- | --- | --- | --- | --- | --- | --- | --- |
| Amphotericin preparation (J02AA) | 8.3 | <0.0001 | 0.7465 (-8.1331 to 9.6261) | 0.8655 | 10.5 | 2.3 | ARMA(1,0) |
| Triazole and tetrazole derivatives (J02AC) | 12.8 | <0.0001 | -1.8875 (-6.1403 to 2.3653) | 0.3875 | 10.7 | 1.7 | ARMA(1,0) |
| Other Antimycotics (J02AX) | 1.8 | <0.0001 | -0.0286 (-1.5291 to 1.4719) | 0.9682 | 1.7 | 0.9 | ARMA(1,0) |
| **Total Antifungals** | 22.8 | <0.0001 | -1.4122 (-11.2265 to 8.4021) | 0.7804 | 22.9 | 0.3 | ARMA(1,0) |
| **WAVE 1 (April 2020- June 2020)** | | | | | | | |
| Amphotericin preparation (J02AA) | 8.3 | <0.0001 | 3.0394 (-4.7675 to 10.8463) | 0.4441 | 10.7 | 3.2 | ARMA(1,0) |
| Triazole and tetrazole derivatives (J02AC) | 12.8 | <0.0001 | 0.9180 (-2.7994 to 4.6354) | 0.6258 | 13.8 | 2.9 | ARMA(1,0) |
| Other Antimycotics (J02AX) | 1.8 | <0.0001 | 1.1825 (-0.1538 to 2.5188) | 0.0866 | 2.8 | 0.6 | ARMA(1,0) |
| **Total Antifungals** | 22.8 | <0.0001 | 5.2675 (-3.6371 to 14.1721) | 0.2463 | 27.3 | 6.2 | ARMA(1,0) |
| **WAVE 2 (July 2020 - April 2021)** | | | | | | | |
| Amphotericin preparation (J02AA) | 8.3 | <0.0001 | 1.8529 (-3.2354 to 6.9412) | 0.4741 | 10.3 | 7.0 | ARMA(1,0) |
| Triazole and tetrazole derivatives (J02AC) | 12.8 | <0.0001 | -1.2842 (-3.7008 to 1.1324) | 0.2976 | 11.3 | 2.1 | ARMA(1,0) |
| Other Antimycotics (J02AX) | 1.8 | <0.0001 | 0.7421 (-0.1432 to 1.6274) | 0.1038 | 2.6 | 1.1 | ARMA(1,0) |
| **Total Antifungals** | 22.8 | <0.0001 | 1.3431 (-4.5976 to 7.2838) | 0.6542 | 24.2 | 8.0 | ARMA(1,0) |
| **WAVE 3 (April 2021 - September 2022)** | | | | | | | |
| Amphotericin preparation (J02AA) | 8.3 | <0.0001 | -0.8618 (-5.1436 to 3.4200) | 0.6905 | 7.2 | 4.6 | ARMA(1,0) |
| Triazole and tetrazole derivatives (J02AC) | 12.8 | <0.0001 | 0.1400 (-1.8877 to 2.1677) | 0.8891 | 13.0 | 2.8 | ARMA(1,0) |
| Other Antimycotics (J02AX) | 1.8 | <0.0001 | -0.6658 (-1.4162 to 0.0846) | 0.0848 | 1.1 | 1.0 | ARMA(1,0) |
| **Total Antifungals** | 22.8 | <0.0001 | -1.3275 (-6.3478 to 3.6928) | 0.6048 | 21.3 | 4.3 | ARMA(1,0) |

**Consumption of alternative treatment offered as anti-COVID during the all-wave of the COVID-19 pandemic period.**

**Table S4: Corticosteroid consumption during all waves of the COVID-19 pandemic period (February 2020 to September 2022).**

| **Transition Period (February 2020-March 2020)** | **Constant** | **p-value** | **Coefficient (95% CI)** | **p-value** | **Mean** | **SD** | **Noise Model** |
| --- | --- | --- | --- | --- | --- | --- | --- |
| Dexamethasone (H02AB02) | 563.5 | <0.0001 | -48.0042 (-196.8243 to 100.8159) | 0.5244 | 546.5 | 7.2 | ARMA(1,0) |
| Hydrocortisone (H02AB09) | 82.3 | <0.0001 | 30.6503 (5.7422 to 55.5584) | 0.0183 | 113.0 | 6.5 | ARMA(0,0) |
| Prednisolone (H02AB06) | 316.5 | <0.0001 | -23.6921 (-79.3091 to 31.9249) | 0.4040 | 321.1 | 32.8 | ARMA(1,0) |
| Total corticosteroid | 963.9 | <0.0001 | -41.8878 (-200.8185 to 117.0429) | 0.6048 | 980.5 | 46.5 | ARMA(1,0) |
| **WAVE 1 (April 2020- June 2020)** | | | | | | | |
| Dexamethasone (H02AB02) | 563.5 | <0.0001 | -30.7092 (-176.7632 to 115.3448) | 0.6832 | 434.7 | 209.7 | ARMA(1,0) |
| Hydrocortisone (H02AB09) | 82.3 | <0.0001 | 11.3966 (-9.2214 to 32.0146) | 0.2797 | 93.7 | 28.5 | ARMA(0,0) |
| Prednisolone (H02AB06) | 316.5 | <0.0001 | -88.1549 (-142.9930 to -33.3168) | 0.0023 | 231.0 | 48.0 | ARMA(1,0) |
| Total corticosteroid | 963.9 | <0.0001 | -112.8134 (-270.8748 to 45.2480) | 0.1633 | 759.4 | 237.9 | ARMA(1,0) |
| **WAVE 2 (July 2020 - April 2021)** | | | | | | | |
| Dexamethasone (H02AB02) | 563.5 | <0.0001 | 42.6450 (-66.0884 to 151.3784) | 0.4441 | 624.3 | 205.4 | ARMA(1,0) |
| Hydrocortisone (H02AB09) | 82.3 | <0.0001 | 22.4478 (10.1329 to 34.7627) | 0.0006 | 104.8 | 23.7 | ARMA(0,0) |
| Prednisolone (H02AB06) | 316.5 | <0.0001 | -102.3015 (-141.8859 to -62.7171) | <0.0001 | 207.0 | 40.4 | ARMA(1,0) |
| Total corticosteroid | 963.9 | <0.0001 | -34.5046 (-152.5744 to 83.5652) | 0.5639 | 936.1 | 197.5 | ARMA(1,0) |
| **WAVE 3 (April 2021 - September 2022)** | | | | | | | |
| Dexamethasone (H02AB02) | 563.5 | <0.0001 | -128.2729 (-223.9562 to -32.5896) | 0.0104 | 433.4 | 61.9 | ARMA(1,0) |
| Hydrocortisone (H02AB09) | 82.3 | <0.0001 | 21.1615 (10.9928 to 31.3302) | 0.0001 | 103.5 | 16.9 | ARMA(0,0) |
| Prednisolone (H02AB06) | 316.5 | <0.0001 | -97.1610 (-131.9171 to -62.4049) | <0.0001 | 223.5 | 25.3 | ARMA(1,0) |
| Total corticosteroid | 963.9 | <0.0001 | -211.6036 (-315.3259 to -107.8813) | 0.0002 | 760.3 | 82.6 | ARMA(1,0) |

**Table S5: Remdesivir (antiviral) consumption during all waves of the COVID-19 pandemic (February 2020 to September 2022).**

| **Transition Period (February 2020-March 2020)** | **Constant** | **p-value** | **Coefficient (95% CI)** | **p-value** | **Mean** | **SD** | **Noise Model** |
| --- | --- | --- | --- | --- | --- | --- | --- |
| Remdesivir (J05AB16) | -0.001 | 0.9970 | 0.0699 (-5.1610 to 5.3008) | 0.9762 | 0.0 | 0.0 | ARMA(3,1) |
| **WAVE 1 (April 2020- June 2020)** | | | | | | | |
| Remdesivir (J05AB16) | -0.001 | 0.9970 | 1.5285 (-2.5882 to 5.6452) | 0.4680 | 0.1 | 0.2 | ARMA(1,3) |
| **WAVE 2 (July 2020 - April 2021)** | | | | | | | |
| Remdesivir (J05AB16) | -0.001 | 0.9970 | 6.9457 (5.0074 to 8.8840) | <0.0001 | 6.8 | 11.1 | ARMA(3,1) |
| **WAVE 3 (April 2021 - September 2022)** | | | | | | | |
| Remdesivir (J05AB16) | -0.001 | 0.9970 | 2.5785 (1.1737 to 3.9833) | 0.0006 | 3.0 | 2.2 | ARMA(3,1) |

**Table S6: Monoclonal antibodies (mABs) consumption during all waves of the COVID-19 pandemic (February 2020 to September 2022).**

| **Transition Period (February 2020-March 2020)** | **Constant** | **p-value** | **Coefficient (95% CI)** | **p-value** | **Mean** | **SD** | **Noise Model** |
| --- | --- | --- | --- | --- | --- | --- | --- |
| Tocilizumab (L04AC07) | 23.7 | <0.0001 | -2.9479 (-20.9738 to 15.0780) | 0.7500 | 16.1 | 2.9 | ARMA(1,0) |
| Sarilumab (L04AC14) | 0.0 | 0.9999 | 0.0000 (-9.7381 to 9.7381) | 0.9999 | 1.0 | 1.0 | ARMA(0,0) |
| Baricitinib (L04AA37) | 0.0 | 0.9999 | 0.0000 (-0.4870 to 0.4870) | 0.9999 | 1.0 | 1.0 | ARMA(0,0) |
| Sotrovimab (J06BD05) | 0.0 | 0.9999 | 0.0000 (-0.0319 to 0.0319) | 0.9999 | 1.0 | 1.0 | ARMA(0,0) |
| **WAVE 1 (April 2020- June 2020)** | | | | | | | |
| Tocilizumab (L04AC07) | 23.7 | <0.0001 | 6.5425 (-12.0137 to 25.0987) | 0.4927 | 26.0 | 3.7 | ARMA(1,0) |
| Sarilumab (L04AC14) | 0.0 | 0.9999 | 0.0000 (-8.0608 to 8.0608) | 0.9999 | 0.0 | 0.0 | ARMA(0,0) |
| Baricitinib (L04AA37) | 0.0 | 0.9999 | 0.0000 (-0.4031 to 0.4031) | 0.9999 | 0.0 | 0.0 | ARMA(0,0) |
| Sotrovimab (J06BD05) | 0.0 | 0.9999 | 0.0000 (-0.0264 to 0.0264) | 0.9999 | 0.0 | 0.0 | ARMA(0,0) |
| **WAVE 2 (July 2020 - April 2021)** | | | | | | | |
| Tocilizumab (L04AC07) | 23.7 | <0.0001 | 8.7290 (-6.2243 to 23.6823) | 0.2544 | 35.0 | 26.5 | ARMA(1,0) |
| Sarilumab (L04AC14) | 0.0 | 0.9999 | 0.0000 (-4.8147 to 4.8147) | 0.9999 | 0.0 | 0.0 | ARMA(0,0) |
| Baricitinib (L04AA37) | 0.0 | 0.9999 | 0.2438 (0.0031 to 0.4845) | 0.0507 | 0.2 | 0.5 | ARMA(0,0) |
| Sotrovimab (J06BD05) | 0.0 | 0.9999 | 0.0000 (-0.0158 to 0.0158) | 0.9999 | 0.0 | 0.0 | ARMA(0,0) |
| **WAVE 3 (April 2021 - September 2022)** | | | | | | | |
| Tocilizumab (L04AC07) | 23.7 | <0.0001 | 0.6056 (-12.9482 to 14.1594) | 0.9286 | 24.4 | 12.6 | ARMA(1,0) |
| Sarilumab (L04AC14) | 0.0 | 0.9999 | 7.2029 (3.2272 to 11.1786) | 0.0007 | 7.2 | 13.8 | ARMA(0,0) |
| Baricitinib (L04AA37) | 0.0 | 0.9999 | 0.3839 (0.1851 to 0.5827) | 0.0003 | 0.4 | 0.6 | ARMA(0,0) |
| Sotrovimab (J06BD05) | 0.0 | 0.9999 | 0.0232 (0.0102 to 0.0362) | 0.0008 | 0.0 | 0.0 | ARMA(0,0) |

**Table S7: AWaRe antibiotic consumption during all waves of the COVID-19 pandemic (February 2020 to September 2022).**

| **Transition Period (February 2020-March 2020)** | **Constant** | **p-value** | **Coefficient (95% CI)** | **p-value** | **Mean** | **SD** | **Noise Model** |  |
| --- | --- | --- | --- | --- | --- | --- | --- | --- |
| Access % | 0.6 | <0.0001 | 0.0284 (-0.0037 to 0.0605) | 0.0848 | 0.62 | 0.03 | ARMA(1,0) |  |
| Watch % | 0.4 | <0.0001 | -0.0249 (-0.0570 to 0.0072) | 0.1309 | 0.38 | 0.03 | ARMA(1,0) |  |
| Reserve % | 0.0 | <0.0001 | 0.0008 (-0.0049 to 0.0065) | 0.7880 | 0.01 | 0.00 | ARMA(1,0) |  |
| **WAVE 1 (April 2020- June 2020)** | | | | | | | | |
| Access % | 0.6 | <0.0001 | -0.0088 (-0.0435 to 0.0259) | 0.6188 | 0.59 | 0.05 | ARMA(1,0) |  |
| Watch % | 0.4 | <0.0001 | 0.0157 (-0.0182 to 0.0496) | 0.3610 | 0.40 | 0.05 | ARMA(1,0) |  |
| Reserve % | 0.0 | <0.0001 | -0.0045 (-0.0110 to 0.0020) | 0.1785 | 0.00 | 0.00 | ARMA(1,0) |  |
| **WAVE 2 (July 2020 - April 2021)** | | | | | | | | |
| Access % | 0.6 | <0.0001 | -0.0081 (-0.0347 to 0.0185) | 0.5506 | 0.61 | 0.02 | ARMA(1,0) |  |
| Watch % | 0.4 | <0.0001 | 0.0049 (-0.0191 to 0.0289) | 0.6905 | 0.38 | 0.02 | ARMA(1,0) |  |
| Reserve % | 0.0 | <0.0001 | 0.0014 (-0.0047 to 0.0075) | 0.6471 | 0.01 | 0.00 | ARMA(1,0) |  |
| **WAVE 3 (April 2021 - September 2022)** | | | | | | | | |
| Access % | 0.6 | <0.0001 | -0.0020 (-0.0262 to 0.0222) | 0.8734 | 0.61 | 0.01 | ARMA(1,0) |  |
| Watch % | 0.4 | <0.0001 | -0.0026 (-0.0239 to 0.0187) | 0.8111 | 0.37 | 0.02 | ARMA(1,0) |  |
| Reserve % | 0.0 | <0.0001 | 0.0021 (-0.0038 to 0.0080) | 0.4989 | 0.01 | 0.00 | ARMA(1,0) |  |

**Figure S1: Trend of antibiotic consumption during the study period from April 2017 to September 2022**

*Antibiotic consumptions were calculated as DDDs and adjusted to 1000 occupied per day. All antibiotics included are classified as WHO **J01** only for systematic use only.

**Figure S2: Percentage (%) consumption of antibiotics according to WHO AWaRe Classification during the study period from April 2017 to September 2022**

*During the first wave, the percentage of watch category antibiotics was raised
